# Combined Therapy with Shock Wave and Retrograde Bone Marrow-Derived Cell Transplantation for Osteochondral Lesions of the Talus

**DOI:** 10.1038/s41598-017-02378-9

**Published:** 2017-05-18

**Authors:** Fuqiang Gao, Na Chen, Wei Sun, Bailiang Wang, Zhencai Shi, Liming Cheng, Zirong Li, Wanshou Guo

**Affiliations:** 1 Centre for Osteonecrosis and Joint-Preserving & Reconstruction, Department of Orthopedic Surgery, Beijing Key Laboratory of Arthritic and Rheumatic Diseases, China-Japan Friendship Hospital, National Health and Family Planning Commission of the People’s Republic of China, Beijing, 100029 China; 2 Department of Internal Medicine, Peking University Civil Aviation General Hospital, Beijing, 100123 China

## Abstract

Multiple treatment strategies have been developed for osteochondral lesions (OCLs) of the talus. The purpose of this retrospective study was to assess retrograde autologous bone marrow cell (BMC) transplantation via core drilling (CD) combined with focused extracorporeal shock wave treatment (ESWT) in undisplaced OCL of the talus. A total of 69 patients with unilateral osteochondral lesions of the talus (Hepple grade I–III) were divided into two groups: 41 patients received combined therapy of ESWT and BMC transplantation (group A), while 28 were administered BMC transplantation alone (group B). The patients were followed up clinically and radiographically for a minimum of 2 years. Mean follow-up was 4.1 ± 2.8 years. AOFAS scores increased more significantly while pain intensity levels decreased in group A after treatment, compared with group B values (*P* < 0.001). In MRI follow-up, a more remarkable improvement of OCLs of the talus was observed in group A compared with group B (*P* = 0.040). Therefore, the combined technique reported here is a highly effective therapeutic option in OCLs of the talus with intact cartilage. It promotes patient recovery with pain control, and improves clinical outcome for more than 2 years after surgery.

## Introduction

Multiple treatment strategies have been developed for osteochondral lesions (OCLs) of the talus^1^. However, it is absolutely essential to revitalize the subchondral bone of OCLs in the therapeutic rationale^2^. This can be achieved by several techniques, including debridement, micro-fracturing, anterograde drilling, osteochondral transplantation, and bone grafting, depending on the local cartilage condition, which have been recommended for broken cartilage surfaces and unstable lesions^3–7^. In contrast, retrograde drilling (RD) deals with subchondral necrotic sclerotic lesions for bone marrow stimulation without damaging the cartilage surface in the treatment of undisplaced OCLs of the talus^2, 8–10^. Several studies have reported the usefulness to supply autologous bone marrow-derived cells (BMDCs) in the subchondral bone to the lesions via arthroscopic anterograde drilling, which may regenerate the damaged subchondral lesions^11–13^. However, the traditional mode of arthroscopic drilling can injure the intact tibial cartilage and undetached talar cartilage, when the Kirschner wire is inserted into the lesion^8, 10^. Therefore, autologous bone marrow-derived cell implantation via retrograde core drilling, which can prevent articular cartilage injury, may constitute the future trend for treating undisplaced OCLs of the talus. Recent studies revealed that extracorporeal shock wave therapy (ESWT) induces neovascularization, and upregulates angiogenesis and osteogenesis-related growth factors^14, 15^. ESWT has shown efficacy in the treatment of certain orthopedic conditions, such as long-bone nonunion fractures^16, 17^. Lyon *et al*.^18^ claimed that ESWT accelerates the healing rate, and improves the cartilage and subchondral bone quality in a rabbit model of OCL in the early stage. A clinical trial^19^ demonstrated that ESWT results in considerable improvement of early stage OCLs of the talus, effectively relieving pain and improving ankle function. Moreover, OCLs of the talus have been considered an indication of ESWT by the International Society for Medical Shockwave Treatment (ISMST) since March 2008^20^.

However, no reports on clinical results of BMDC via RD combined with ESWT are available. It can be speculated that combined operative procedures may be more effective than single BMDC transplantation for OCLs of the talar dome without cartilage detachment. In this study, we retrospectively evaluated the effectiveness of retrograde autologous BMDC transplantation combined with fluoroscopy-guided focused ESWT in undisplaced OCLs of the talus. We hypothesized that the combined technique would improve pain relief and talus function, without substantial complications compared with the single BMDC transplantation method.

## Results

Between May 2010 and June 2014, 69 patients with OCLs of the talus were first treated by fluoroscopy-guided retrograde core drilling and autologous BMDC transplantation combined with focused ESWT. OCLs of the talar dome were diagnosed preoperatively by radiography and MRI. Inclusion criteria were symptomatic undisplaced OCLs of the talus, and grade I to III according to the MRI radiological classification of Hepple^8^. Exclusion criteria were: >grade IV lesions; maximal lesion area >1.5 cm^2^ in MR cross-sectional axial planes of the talar dome^21^; osteoarthritis or kissing lesions of the ankle, rheumatoid arthritis, ligamentous instability at clinical examination, and a history of ankle fracture. The patients were assigned to receive retrograde CD and autologous BMDC transplantation following ESWT (group A) (n = 41) and BMDC transplantation alone (group B) (n = 28), respectively. The characteristics of all patients included in this study are shown in Table 1. There were 19 Hepple Grade I patients (19 hips), 42 Grade II cases (42 hips), and 8 Grade III patients (8 hips). All lesions were considered to be chronic, with persistent symptoms for over three months and unsuccessful conservative treatment. The study population consisted of 32 female and 37 male patients, with a total of 69 talar lesions. Average age was 46.2 years (19–62 years); BMI (Body Mass Index) values were 25.1 ± 4.9 kg/m^2^. The mean maximal lesion area measured in MR cross-sectional axial planes of the talar domes was 1.1 cm^2^, ranging from 0.7 to 1.4 cm^2^ (i.e. all <1.5 cm^2^). There were 61 medial and 8 lateral lesions. The patients with lesions localized at the lateral talus had trauma. There were 23 patients with no evidence of trauma among the 61 cases with lesions at the medial talus. The follow-up period was 4.1 ± 2.8 years.

**Table 1:** Patients characteristics.

Characteristics	Group A (n = 41)	Group B (n = 28)	*P* value
Female, n (%)	17 (41.5)	15 (53.6)	0.322
Age, years	43.1 ± 8.2	47.4 ± 9.4	0.371
BMI, kg/m^2^	24.9 ± 4.6	25.3 ± 5.1	0.890
IBST, weeks	6.6 ± 3.1	6.9 ± 2.9	0.162
Duration of follow-up, years	3.7 ± 1.2	4.3 ± 2.2	0.113
Trauma History, (%)	26 (63.4)	19 (67.8)	0.704
**Hepple MRI Grade**			
Grade I	11	8	0.874
Grade II	24	18	
Grade III	6	2	

Note: BMI:Body Mass Index; IBST: Interval between the onset of symptoms and the beginning of treatment.

### Clinical outcomes

**Figure 1:**
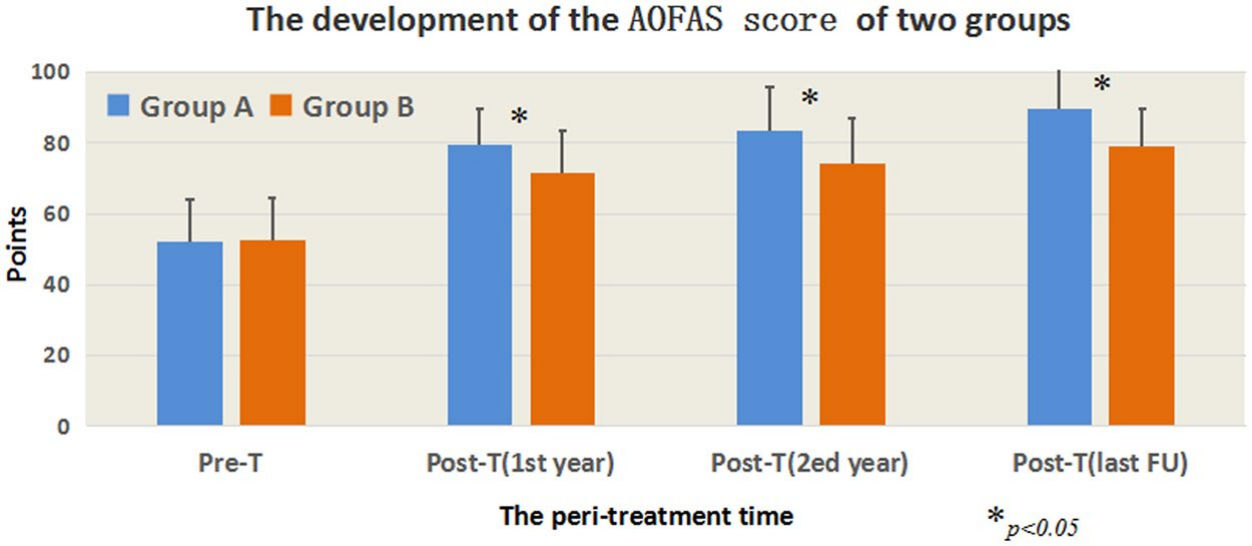
Progression of AOFAS scores in peri-treatment between the two groups.

The American Orthopedic Foot and Ankle Society (AOFAS) scores and subscales in both groups are shown in Table 2. The overall AOFAS score was increased significantly in both groups at final follow-up (*P* < 0.001). This might be mainly caused by pain relief. In both groups, the patients reported pain relief post-treatment at final follow-up (*P* < 0.001); function sub-scores were increased slightly after removal of the pain score, with statistically significant difference (*P* < 0.001). No significant difference was detected in the alignment subscale (*P* > 0.05). However, all patients described the daily life function as significantly improved. Compared with group B individuals, group A showed higher and earlier improvement in pain and function scales in the AOFAS score at final follow-up after therapeutic intervention (*P* < 0.05). Moreover, AOFAS scores of group A patients continued to improve more overtly over the follow-up period compared with group B values (Fig. 1). Significant improvement of AOFAS scores was observed in group A, from 51.9 ± 11.9 to 79.4 ± 10.3 points (first year), 83.6 ± 12.0 (second year), and 89.5 ± 11.7 points (final follow-up) after therapeutic intervention. Gradual improvement in the VAS was observed in group B, from 52.3 ± 12.1 to 71.7 ± 11.8 points (first year), 74.2 ± 12.9 points (second year) and 79.1 ± 10.6 points (final follow-up) after therapeutic intervention. The differences between the two groups at these time points were statistically significant (*P* < 0.05).

**Table 2:** The results of the subscales of American Orthopaedic Foot and Ankle Society (AOFAS) score.

Subscales	Pain scale	Function scale	Alignment scale	Total
** Group A** (**n** = **41**)				
Pre-treatment	9.2 ± 8.3	32.5 ± 9.5	9.3 ± 1.7	51.9 ± 11.9
Post-treatment (At the last FU)	35.4 ± 10.8	44.8 ± 10.1	9.3 ± 1.7	89.5 ± 11.7
*P* value	<0.001	<0.001	0.999	<0.001
** Group B** (**n** = **28**)				
Pre-treatment	9.1 ± 9.4	33.9 ± 9.6	9.3 ± 1.7	52.3 ± 12.1
Post-treatment (At the last FU)	29.1 ± 11.9	40.7 ± 7.8	9.3 ± 1.8	79.1 ± 10.6
* P* value	<0.001	<0.001	0.998	<0.001

Note: FU: follow-up.

Patients in each group were divided into subgroups by trauma history and the Hepple MRI grade (Table 3). In both groups, grade I and II lesions showed improved results compared with grade III counterparts. However, no statistically significant differences among grade I, II and III lesions were observed at final follow-up (*P* > 0.05). Patients with a history of ankle trauma in group A showed slightly improved AOFAS scores than those without (*P* = 0.046). However, similar AOFAS scores were found in group B (*P* = 0.072).

**Table 3:** The results of American Orthopaedic Foot and Ankle Society (AOFAS) score and its variation after treatment by trauma history and MRI grade within each group.

Parameter	n	Pre-treatment	Post-treatment	Variation	*P*_3_ value
**Group A** (**n** = **41**)					
Hepple MRI Grade					
Grade I	11	53.4 ± 10.1	91.3 ± 12.6	38.1 ± 11.9	0.634
Grade II	24	50.2 ± 12.6	88.5 ± 10.2	37.9 ± 10.4	
Grade III	6	48.9 ± 8.4	87.8 ± 9.1	38.3 ± 12.1	
*P*_*1*_ value		0.073	0.068		
Trauma History					
Trauma	26	49.3 ± 13.1	90.7 ± 12.8	41.3 ± 12.2	0.046
No trauma	15	52.7 ± 10.9	87.9 ± 11.9	36.1 ± 10.9	
*P*_*2*_ value		0.049	0.103		
** Group B** (**n** = **28**)					
Hepple MRI Grade		52.3 ± 12.1	79.1 ± 10.6		
Grade I	8	51.1 ± 9.7	82.3 ± 8.1	32.1 ± 9.3	0.297
Grade II	18	53.5 ± 13.6	80.6 ± 12.0	27.7 ± 12.4	
Grade III	2	47.5 ± 2.1	77.0 ± 1.4	29.5 ± 3.5	
*P*_*1*_ value		0.096	0.118		
Trauma History					
Trauma	19	51.9 ± 12.6	82.5 ± 13.3	32.7 ± 12.9	0.072
No trauma	9	52.7 ± 11.7	78.6 ± 11.4	27.3 ± 12.1	
*P*_*2*_ value		0.106	0.074		

Note: *P*_*1*_ value: comparison of data among Hepple MRI Grade groups for AOFAS score of pre-treatment and post-treatment. *P*_*2*_ value: comparison of data between trauma group and no trauma group for AOFAS score of pre-treatment and post-treatment. *P*_*3*_ value: comparison of data by trauma history and MRI grade for peri-treatment AOFAS score variation.

### Radiological outcome

**Figure 2:**
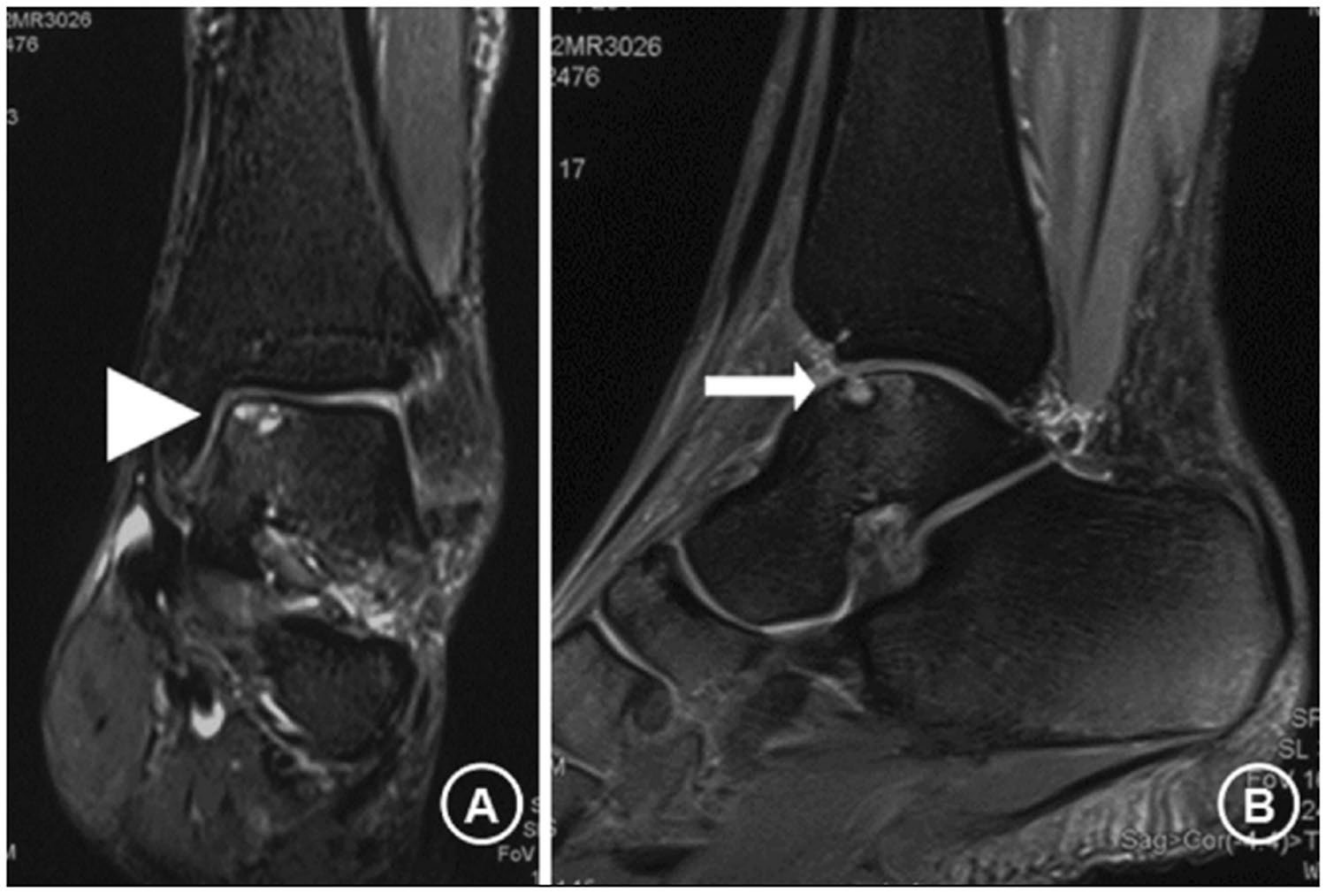
T2-weighted coronal (**A**) and sagittal (**B**) magnetic resonance images of the ankle. The images confirm the presence of subchondral osteochondral lesions (**A**, arrow head; **B**, arrow) of the talus with apparent intact cartilage. Note: The patient consented to publish the specific information.

**Figure 3:**
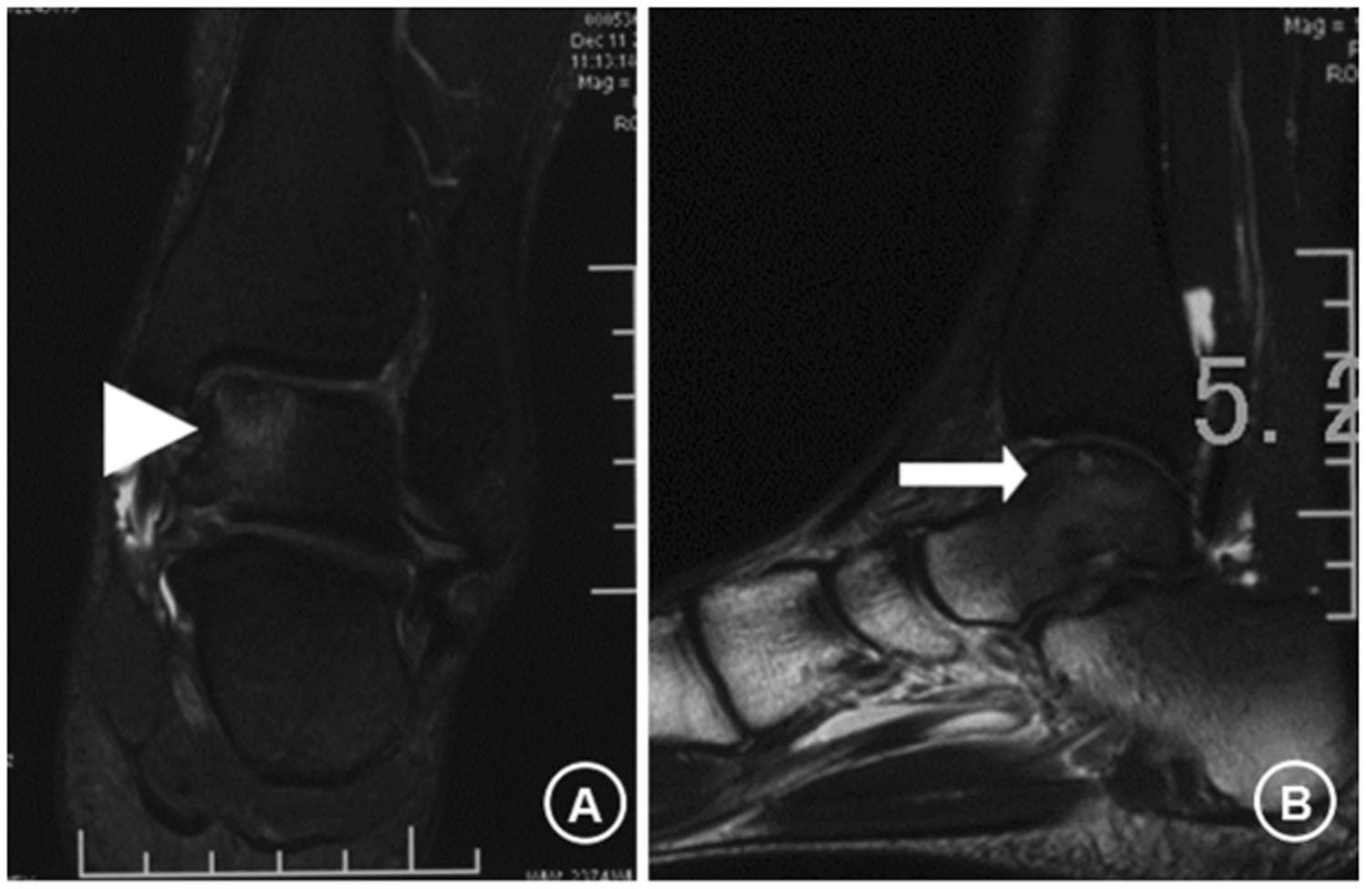
T2-weighted coronal (**A**) and sagittal (**B**) magnetic resonance images of the ankle. Images at 6 months post-retrograde autologous bone marrow cell transplantation via core drilling combined with focused extracorporeal shock wave treatment. Clear evidence of mild marrow edema and repair of osteochondral lesions (**A**, arrow head; **B**, arrow). Note: The patient consented to publish the specific information.

MRI findings demonstrated progressive regression of the OCLs in both groups. Improvement of MRI findings was observed in 62 cases (89.9%) (Figs 2 and 3). These patients showed no intact cartilage surface, with complete subchondral bone remodeling in MRI follow up. MRI scans showed that group A had higher incidence of distinct reduction of OCLs at 1 year compared with group B (38 cases, 92.7% vs. 21 cases, 75.0%; *P* = 0.040). MRI at final follow-up showed OCL reduction in 95.1% (39/41) group A patients, for 82.1% (23/28) in group B. However, MRI results at final follow-up were not significantly different (*P* = 0.080). Final failure of OCL treatment might be associated with large bone cartilage damage and partially separated or detached fragments. At final follow-up, no joint space narrowing, necrosis or stress fracture of the talus, or compression of the OCL area was found.

### Side-effects

Five patients in group A and seven in group B complained of ankle swelling or creaky bone for up to 12 weeks postoperatively. One case of minor hypesthesia of the midfoot was recorded in group B, and may be associated with cutaneous nerve injury. No delayed wound healing, ankle joint infection, or deep vein thrombosis cases were recorded. No clinically detectable neuromuscular, systemic, and device-related adverse effects were recorded; no reoperations were carried out in either group.

## Discussion

Preserving an intact cartilage stratum is the therapeutic principle in treating stable lesions; hence, retrograde drilling techniques should be recommended in such cases^2^. Studies showed that retrograde drilling can manage subchondral necrotic sclerotic lesions for bone marrow stimulation without damaging the cartilage surface in the treatment of undisplaced OCLs of the talus^2, 8–10^. These lesions can be excised accurately under the guidance of fluoroscopy^10^, MRI^22^ or computer-assisted navigation^23–25^. Arthroscopic-controlled or computer-assisted navigation could allow a more exact localization. However, this requires more complex and expensive hardware support^26, 27^. In this study, fluoroscopy-guided retrograde drilling techniques implemented by a group of experienced doctors were selected. Kono *et al*.^10^ revealed that compared with transmalleolar drilling, retrograde drilling for osteochondral lesions of the talar dome yields superior results, while assessing 30 patients with undetached lesions. Mid-term follow-up also showed successful results. Indeed, Schuman *et al*.^28^ found 82% good to excellent outcomes following drilling at an average of 4.8-year follow-up. A recent systematic review found an overall success rate of 85%^29^. Several studies reported the usefulness of supplying autologous bone marrow-derived cells (BMDCs) in the subchondral bone to lesions via arthroscopic anterograde drilling, which may regenerate the damaged subchondral lesions^1, 11–13, 27^. Buda *et al*.^11^ stated that use of BMDCs supported by scaffolds to repair osteochondral lesions results in significant clinical improvement of the talus, maintained over time. Clinical results peaked at 24 months, before declining gradually to settle at an AOFAS score of around 80 at a maximum follow-up of 72 months^11^. Meanwhile, histological follow-up by Giannini *et al*.^30^ found that injection of bone marrow aspirates can regenerate the tissue to various degrees in talar osteochondral lesion remodeling, improving function scores and overcoming the drawbacks of previous techniques. Buda *et al*.^13^ compared autologous chondrocyte implantation (ACI) and BMDC transplantation in the treatment of OCLs. BMDC transplantation was shown to be preferred over ACI in the single step procedure, patient discomfort, and cost. However, the traditional mode of arthroscopic drilling can injure the intact tibial cartilage as well as undetached talar cartilage, when the Kirschner wire is inserted into the lesion^8, 10^. Therefore, autologous BMDCs via retrograde core drilling, which prevents articular cartilage injury, may constitute the future development trend for treating undisplaced OCLs of the talus.

ESWT has shown efficacy in the treatment of certain orthopedic conditions, such as long-bone nonunion fractures^16, 17^. Recent studies revealed that extracorporeal shock wave therapy (ESWT) induces neovascularization, and upregulates angiogenesis and osteogenesis-related growth factors^14, 15^. A clinical trial^19^ demonstrated that ESWT results in considerable improvement in the early stage OCLs of the talus, effectively relieving pain and improving ankle function. Animal experiments^18^ suggest that ESWT accelerates the healing rate, and improves cartilage and subchondral bone quality in a rabbit model of OCL in the early stage. A history of trauma might affect the final AOFAS score. Chuckpaiwong *et al*.^31^ demonstrated that a history of trauma is significantly associated with a poor outcome. However, van Bergen *et al*.^26^ found that traumatic etiology is not associated with the clinical or radiological outcome after microfracture. Meanwhile, the current study found a slight difference in both groups, likely because shock waves can repair post-traumatic reactions in the local bone tissue, which needs to be confirmed in the future.

Studies of retrograde autologous BMDC transplantation combined with focused ESWT in OCLs in the talus are scarce. In this study, we inferred that the combined method would result in more effective pain relief and functional improvement of the affected talus, without substantial complications in undisplaced OCLs of the talus compared with BMDC transplantation as monotherapy. We found that AOFAS scores were increased more significantly while pain intensity was reduced after the combinatory treatment than those of BMDC transplantation as monotherapy (*P* < 0.01). In both groups, the patients reported pain relief at final follow-up (*P* < 0.001); the function sub-score was significantly increased after removal of the pain score (*P* < 0.001), indicating that function improvement was mainly due to reduced pain. In each group, better results in grade I and II lesions were obtained compared with grade III ones. However, no statistically significant differences were found among grade I, II and III lesions at final follow-up (*P* > 0.05). In MRI follow-up, remarkable improvement of MRI findings was obtained in the vast majority of cases, even within 1 year of treatment, especially in patients that underwent combined treatment.

The synergetic effects of the combined techniques, which remain unclear, need to be further explored. Retrograde drilling of the bone marrow core, created in the subchondral bone, can allow mesenchymal stem cells to form fibrocartilage repair tissue, which can migrate into the lesions^1^. Meanwhile, subchondral bone vascularized after drilling stimulates the release of growth factors and cytokines, increasing mesenchymal stem cell (MSC) recruitment^1, 27, 32, 33^. Pluripotent mesenchymal stem cells can aggregate and differentiate into chondrocyte-like cells, and form repair tissue expressing type II collagen in response to growth factors^1, 34, 35^. The inflammatory response following drilling leads to fibrin clot formation to generate the eventual fibrocartilaginous repair tissue, consisting primarily of type-I collagen that is likely to degenerate over time due to inherently different biological and mechanical properties^27, 35^. Moreover, bone marrow aspirates from the ilium contain a variety of MSCs and hematopoietic cells, which are considered a means of improving osteochondral repair^1, 27, 36^. In addition, the harvested constituents may lead to improved remodeling of the subchondral bone as well as higher type-II collagen amounts and proteoglycan differentiation of the fibrocartilage, thereby causing a more hyaline-like tissue with greater durability^34^. Despite good clinical results of ESWT in the treatment of many orthopedic disorders^17^, the underlying mechanism remains relatively unknown. Shock waves selected with appropriate energy and pulse number can stimulate osteogenesis and angiogenesis^14, 16, 37^. Recent animal studies have revealed that ESWT induces neovascularization, upregulates angiogenesis and growth factors, and promotes cell proliferation and osteogenesis^15, 17^. Furthermore, ESWT promotes bone marrow stromal cell (BMSC) differentiation toward osteoprogenitor cells, and induces membrane hyperpolarization and Ras activation to act as an early signal for osteogenesis in human bone marrow stromal cells^16, 37^.

The limitations of this study should be mentioned. Sample size was relatively small, and follow-up relatively short; the results may not necessarily represent long-term outcomes. The patients were retrospectively evaluated. Because OCLs of the talus are relatively uncommon in our setting, it would have been challenging to conduct a randomized controlled trial. The functional improvement of the talus was assessed subjectively using pain and functional scores, with no objective measures utilized. Due to trauma and medical costs, the current patients declined arthroscopy examination employed in previous reports, and accurate assessment of the talus cartilage surface was nearly impossible. Moreover, a qualitative evaluation of the regenerated tissue was performed using T2-weighted MRI, which currently lacks standardization.

In conclusion, our results indicated that minimally invasive retrograde autologous bone marrow cell transplantation via core drilling combined with focused extracorporeal shock wave is a highly effective therapeutic option in undisplaced osteochondral lesions of the talus with intact cartilage. We found that AOFAS scores were increased significantly while pain intensity was reduced after the combinatory treatment. In follow-up, remarkable improvement of MRI findings was observed in the majority of cases, most within 1 year of treatment. This could promote patient recovery, leading to pain control and improved clinical outcome; these effects lasted for more than 2 year post-surgery.

## Material and Methods

This retrospective, non-blinded comparative study was approved by the Institutional Review Board on Human Studies of the Ethical Committee of China-Japan Friendship hospital. The study procedures adhered to the 1975 Declaration of Helsinki. Informed consent was obtained from all patients. Group A patients adopted the treating mode of “Two-session ESWT First Then retrograde autologous BMDC transplantation”.

### Phase 1: Shock wave treatment

**Figure 4:**
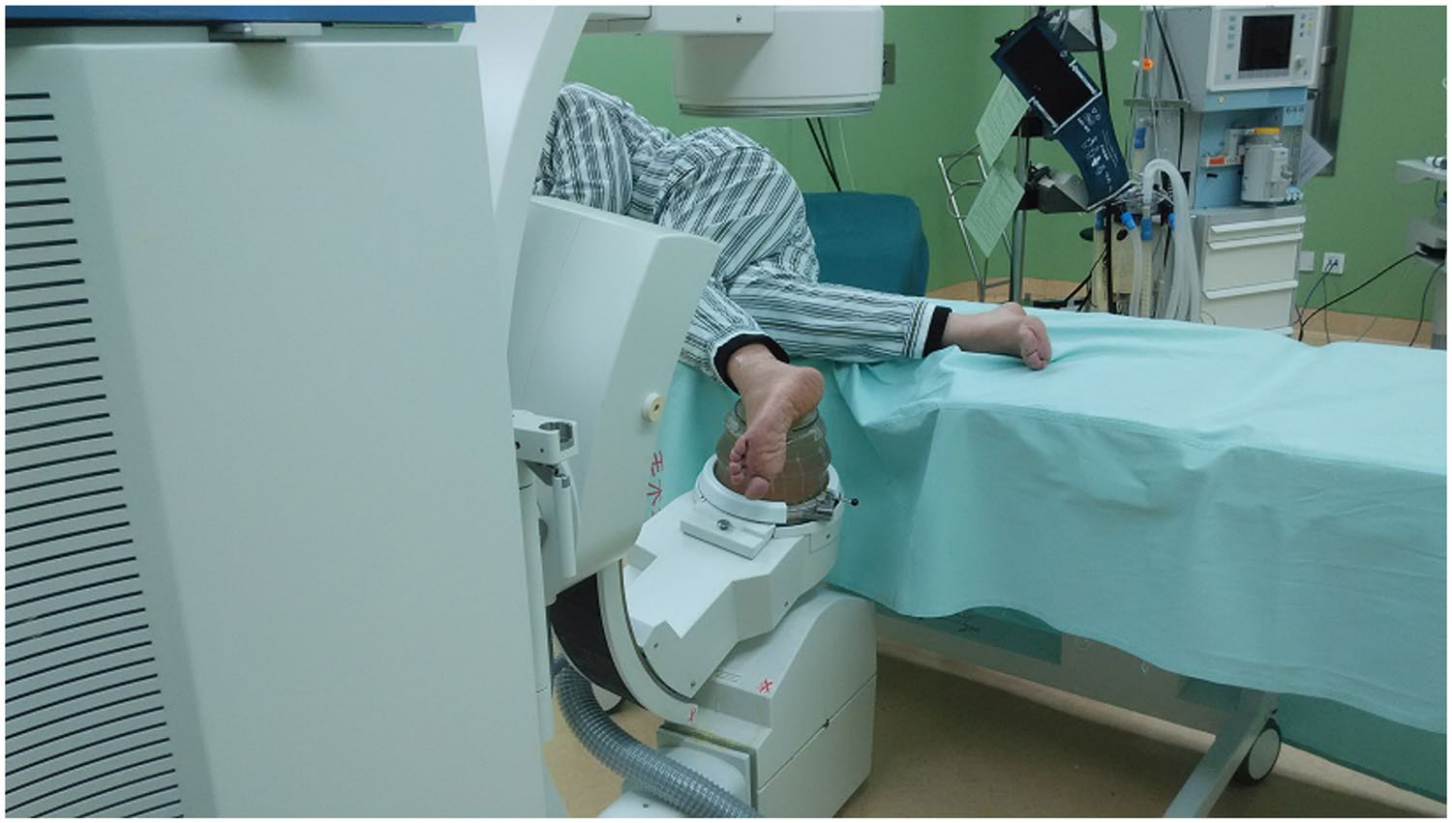
Image showing shockwave treatment of OCLs of the talus (Dornier Compact DELTA II, Munich, Germany). Note: The patient consented to publish the specific information.

Shock wave treatment was applied using an Electromagnetic Shock Wave Emitter (Dornier Compact DELTA II, Munich, Germany) (Fig. 4), with a penetration depth between 0 and 150 mm, and a focus diameter of 4 mm. The shockwave tube generating shock waves was directed to the skin surface near the lesions of the affected ankle. Shock waves were focused around (on the margins of) the talar dome under radiographic guidance. Two to three points were located on the hardened layer around OCLs. The treatment area was prepared with a coupling gel to minimize the loss of shock wave energy at the interface between the device head and skin. All ESWT procedures were performed once with superficial anesthesia by experienced physicians, with the patient in lateral decubitus position on an ESWT table. ESWT orthopedic settings were prepared and used according to previous reports^17, 19, 38^ as follows: number of levels, 1–2; 500–800 pulses at an energy flux density of 0.15–0.28 mJ/mm^2^ (level 1–2) delivered to each lesion; 2000–3000 impulses at a frequency of 2–3 Hz. Each patient underwent two therapy sessions, separated by one week. The applied settings depended on the patient’s condition.

### Phase 2: Retrograde core drilling and autologous BMDC transplantation

**Figure 5:**
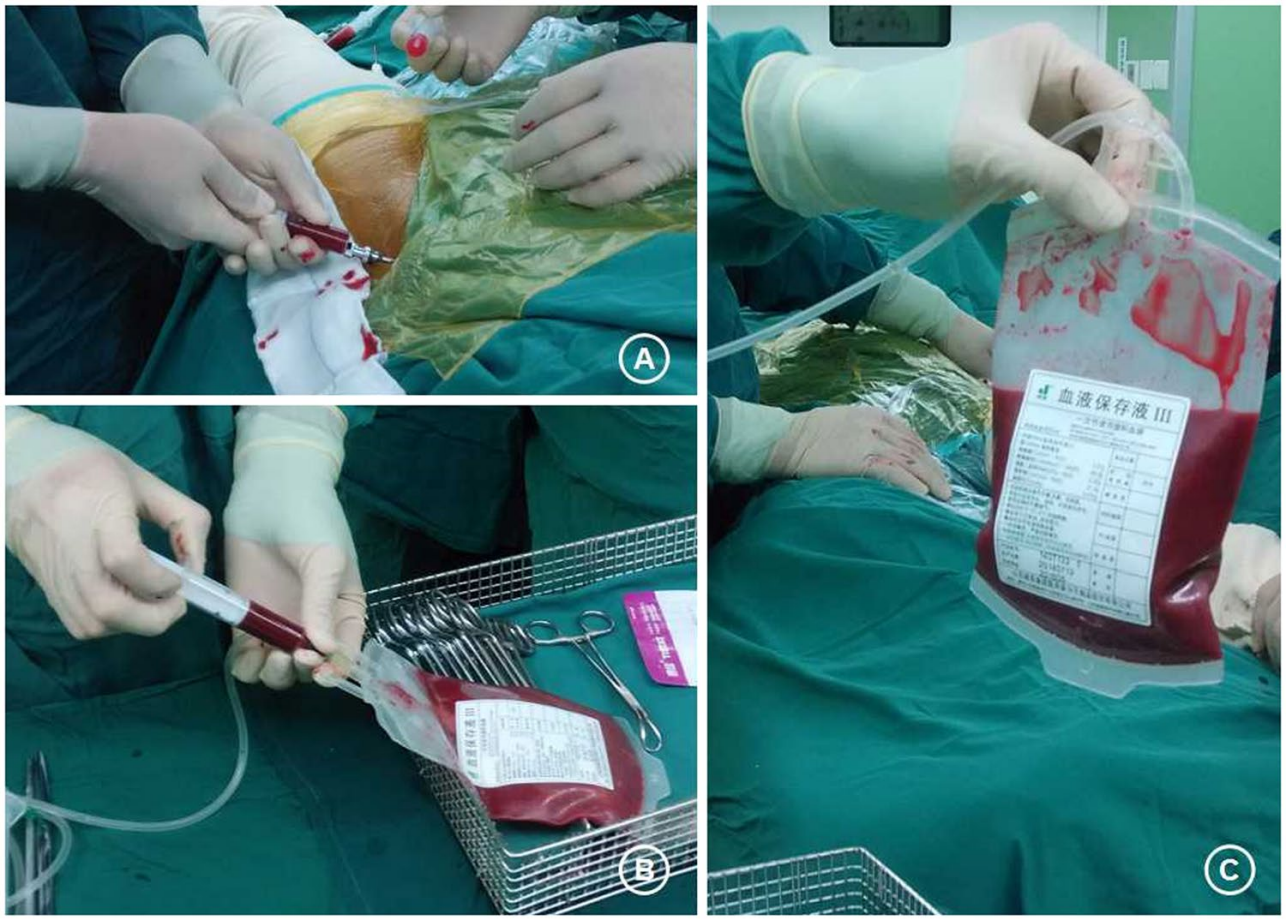
Bone marrow aspirates were harvested in sterile conditions from the anterior iliac crest (**A**), with the patient in the supine position under general or spinal anesthesia. A total of 100–200 ml bone marrow was aspirated per sample (**B,C**).

All patients underwent retrograde autologous BMDC transplantation. BMDCs were harvested as previously described^11–13^. Bone marrow aspirates^30^ were harvested in sterile conditions from the anterior iliac crest, with the patient in the supine position under general or spinal anesthesia (Fig. 5). After insertion of an 11-gauge bone marrow needle 2 cm deep into the spongy bone of the anterior iliac crest, a total of 200–300 ml bone marrow was aspirated into a syringe containing heparin. The needle was rotated 90 degrees clockwise; the whole sequence was repeated, changing the needle location every 3 levels and aspirating another milliliter of marrow during the harvest procedure. This was repeated 4 times. Besides, the bilateral anterior iliac crest was selected if necessary.

**Figure 6:**
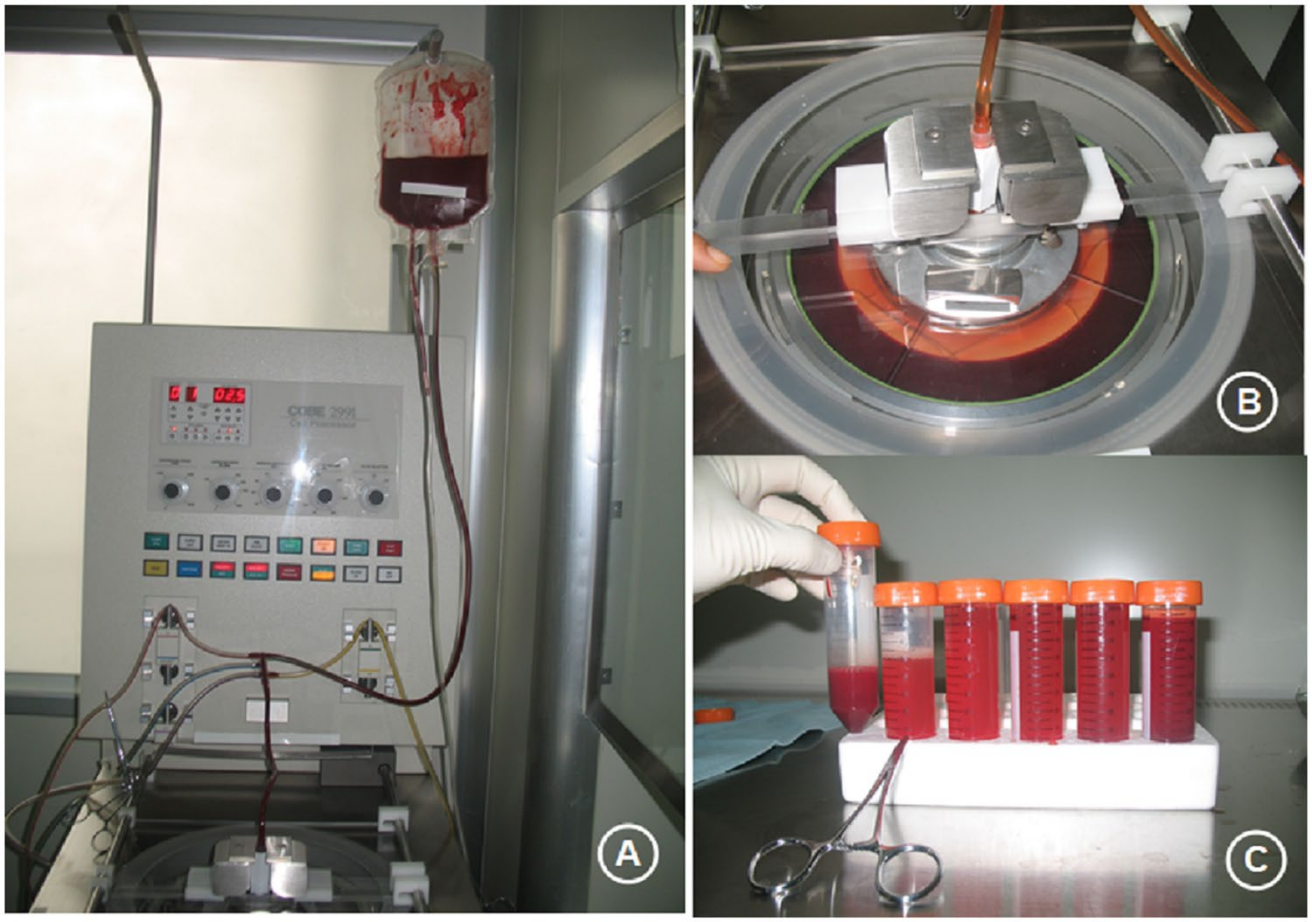
The harvested bone marrow was processed directly in the operating room on a cell concentrator-separator device (**A**, COBE® 2991 Cell Processor, Terumo BCT, Gambro BCT, Inc., Lakewood, USA), spinning at 2000 rpm for 5 minutes (**B**) to separate bone marrow components. These components were stratified according to density and plasma; bone marrow mononuclear cells (BMMCs) and red blood cells were located from the inner to outer layers (**C**). Note: The patient consented to publish the specific information.

The harvested bone marrow was processed directly in the operating room on a cell concentrator-separator device (COBE® 2991 Cell Processor, Terumo BCT, Gambro BCT, Inc., Lakewood, USA), spinning at 2000 rpm for 5 minutes to allow the separation of bone marrow components; the buffy coat deposit was left in the collection chamber. Bone marrow components were stratified according to density and plasma; bone marrow mononuclear cells (BMMCs) and red blood cells were located from the inner to outer layers (Fig. 6). About 30–40 milliliters buffy coat containing MSCs and other nucleated cell populations that constitute the bone marrow microenvironment (monocytes and lymphocytes) were obtained.

**Figure 7:**
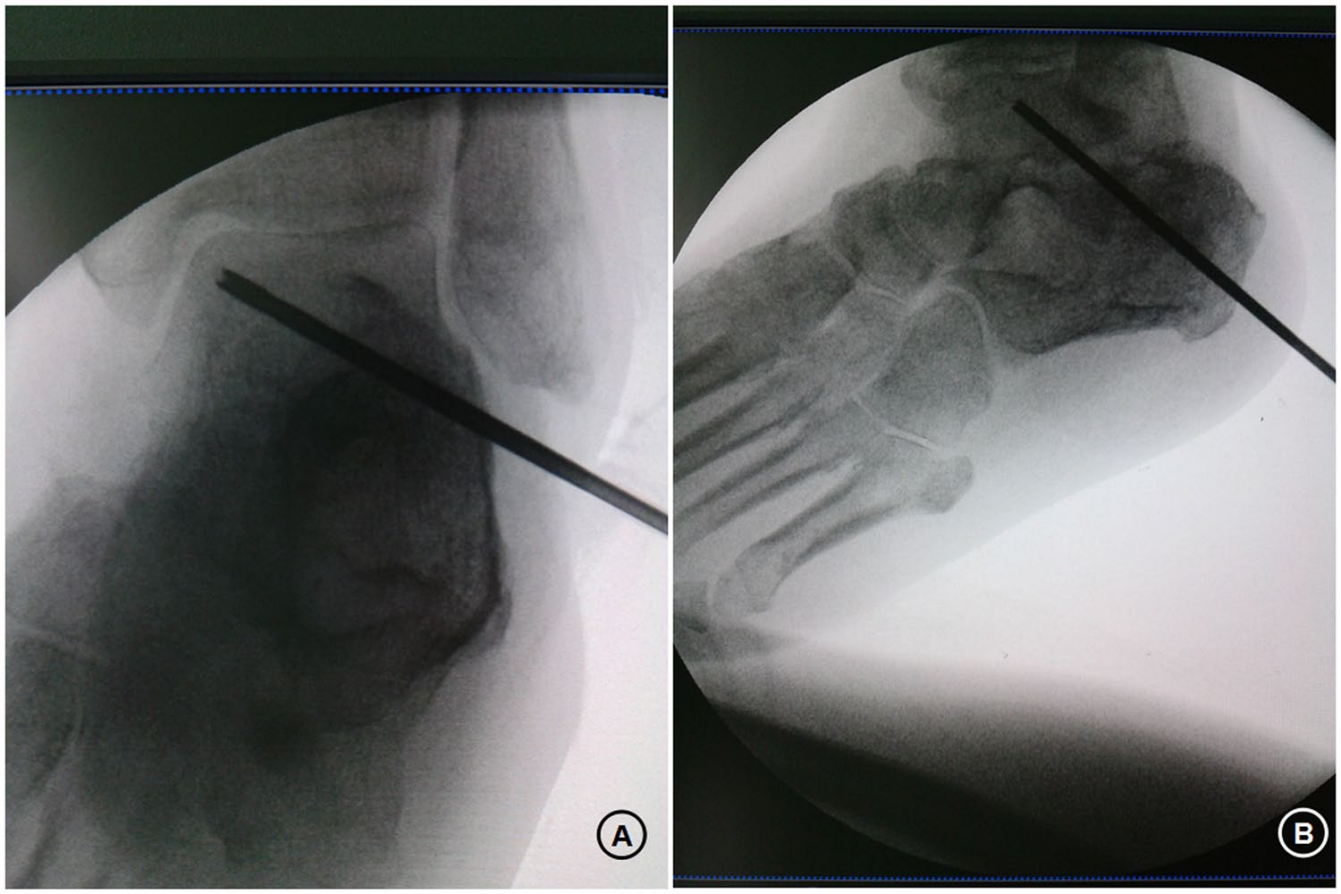
Fluoroscopy-guided positioning of the pilot Kirschner wire into the center of a medial osteochondral lesion (OCL) in AP and lateral plain view radiographs. Guided by the Kirschner wire, the hollow trephine was gently passed through the sclerotic zone of the osteochondral lesion (OCL), controlled by fluoroscopy in AP (**A**) and lateral (**B**) plain view radiographs. Debridement of the osteochondral lesion (OCL) through the drilling hole. The aspirate from the ilium could be injected under the guidance of the hollow trephine to the OCL site. Note: The patient consented to publish the specific information.

Preoperative radiography and MRI were used to evaluate the location of OCLs of the talus. Under fluoroscopic control, a 1.5 mm pilot Kirschner wire was positioned from the opposite talar neck into the subchondral sclerotic zone. Correct aiming was verified in AP and lateral plain view radiographs. Then, the sclerotic zone was gently penetrated by core drilling, with the guide wire carefully advanced into the sclerotic rim of the OCL; the cartilage was not perforated. Afterward, the bone drilling tunnel was enlarged by the hollow trephine under the guidance of a suitable Kirschner wire to ensure the harvest of a cancellous bone cylinder (Fig. 7). Debridement of OCLs through the drilling hole was carried out. Finally, autologous BMDCs harvested from the iliac crest were inserted retrogradely to the lesion through the cancellous bone cylinder. The autologous cancellous bone was driven to protrude and close the drilling core.

### Postoperative management and follow-up

The patients were mobilized with partial weight-bearing and walking aid for 6 weeks; analgesics were provided on demand, with restrictions for impact sports such as sprinting or jumping. The patients were examined clinically and evaluated with relevant scoring systems by a single examiner. AOFAS Ankle-Hindfoot scores^39^ were obtained before treatment, and at 1 month, 3 months, 6 months, 1 year, and 2 years post-treatment. Additionally, MRI with coronal, sagittal and axial planes (T1-and T2-spin-echo sequences, Signa HDx, General Electric Company, USA) was performed before the operation (Fig. 3), and 6 months, 1 year, and 2 years after therapy. Perioperative radiographs and MRI scans were assessed by two experienced radiologists. Clinical outcomes were evaluated by two surgeons.

### Statistical analysis

AOFAS scores were compared before and after the combinatory treatment using paired t-test. Overall clinical outcomes were assessed by Chi-square test for statistical significance, using 95% confidence intervals (95%CI). Comparisons between paired data and independent groups were performed by Student’s t test (normally distributed variables) or rank-sum test (non-normal1y distributed data). Continuous variables were expressed as mean ± standard deviation (SD). Factor analysis of patient age, BMI and follow-up time was carried out using the Pearson product-moment correlation coefficient (r). All statistical analyses were performed with SPSS version 16.0.0 (SPSS; Chicago, IL). P < 0.05 was considered statistically significant.
